# Assessment of maternal and neonatal outcomes in pregnant women with and without gestational diabetes mellitus diagnosed at the three trimesters of pregnancy: a cross-sectional study in a hospital in Northeast Mexico

**DOI:** 10.11604/pamj.2025.50.112.45824

**Published:** 2025-04-25

**Authors:** Rafael Violante-Ortiz, Claudio Requena-Rivera, Francisco Barrera, Norma Fernández-Ordoñez, Alejandra Tavera-Tapia, Jose Eugenio Guerra-Cárdenas, Karla Alejandra Violante-Cumpa, Salvador Mojarro-Bazán, Jorge Rafael Violante-Cumpa

**Affiliations:** 1 Tampico School of Medicine “Dr. Alberto Romo Caballero”, *Universidad Autónoma de Tamaulipas*, Tamaulipas, México,; 2 Center for Metabolic and Cardiovascular Research Studies, Tampico, Tamaulipas, México,; 3 School of Medicine and University Hospital “Dr. José E. González”, *Universidad Autonoma de Nuevo Leon*, Monterrey, Mexico,; 4 Internal Medicine Deparment, University Hospital “Dr. José E. González”, *Universidad Autónoma de Nuevo León*, Monterrey, México,; 5 Endocrinology Division, Internal Medicine Department, University Hospital “Dr. José E. González”, *Universidad Autónoma de Nuevo León*, Monterrey, México

**Keywords:** Gestational diabetes, obesity, gestational diabetes screening

## Abstract

**Introduction:**

gestational diabetes mellitus is a common complication of pregnancy. Diagnostic tests should be performed between 24-28 weeks of pregnancy; however, studies have shown that early or late screening could provide certain benefits in pregnancy outcomes. The study aimed to determine the impact of performing early or late screening tests for GDM in maternal and neonatal outcomes compared to the standard screening time.

**Methods:**

we conducted a cross-sectional study including adult pregnant women with a high risk of GDM. Baseline characteristics, risk factors and differences in maternal and neonatal outcomes were evaluated (e.g. preeclampsia, infections, birth weight, etc.). Multivariable logistic regression analysis was performed to identify factors associated with adverse maternal outcomes and adverse neonatal outcomes.

**Results:**

a total of 803 patients were included, mean gestational age at the end of pregnancy was 37.6+4.4 weeks. 232/803 patients (28.9%) underwent screening in the standard time. A total of 79/232 (34.0%) from those that underwent screening at the standard time were diagnosed with GDM, and 102/286 (35.6%) and 111/285 (38.9%) were diagnosed in the early and late screening times, respectively. Age >30, BMI ≥25, and family history of DM were found as significant risk factors for gestational diabetes mellitus in the multivariable logistic regression analyses. Earlier gestational age, and caesarean section were significantly more frequent in women with GDM compared with women without GDM. No significant differences were found in the neonatal outcomes.

**Conclusion:**

our results suggest a similar proportion of patients are diagnosed with GDM when comparing the three screening times and that early or late screening times do not provide any additional benefits on neonatal outcomes.

## Introduction

Gestational diabetes mellitus (GDM) is a highly prevalent pregnancy-related disease, and its prevalence has been constantly rising through the years [1]. Globally, its prevalence has been reported as high as 10% and as low as 7% [2]. For instance, in the United States in 2018, 7 out of 100 pregnant women were affected by GDM [3]. In contrast, data from 2016 in Mexico suggested that the prevalence of this disease was as high as 30.1% [4,5]. The development of GDM during pregnancy may lead to serious complications that affect both the mother and the child. These complications include preeclampsia, eclampsia, preterm delivery, cesarean delivery, macrosomia, gestational weight gain, and development of type 2 diabetes mellitus (T2DM) after pregnancy [6]. Several risk factors for developing GDM have been described in the literature; these include age >30, overweight or obesity, prior history of GDM or T2DM, and family history of T2DM [7]. Unfortunately, GDM may be misdiagnosed, due to a lack of substantial agreement and high variability in test results [1,8,9].

The current standard guidelines state that an oral glucose tolerance test (OGTT) should be performed in pregnant patients who have a gestational age between 24 and 28 weeks [6]. Previously, the IADPSG recommended using their criteria to diagnose early GDM in patients with high-risk factors, but it was not until 2016 when the same organization stated that these criteria were not applicable for early diagnosis of GDM without providing any other criteria for this manner. Due to the inexistence of valid and applicable criteria and the rate of undiagnosed risk factors, early screening is not being efficient [10]. Therefore, the inclusion of early screening for presumptively healthy patients may assist in the reduction of incidence and prevalence of GDM. Previous studies have shown that early screening and treatment of GDM benefit greatly the pregnancy by reducing the gestational weight gain [7], which is highly associated with maternal and neonatal morbidity/mortality and an excessive weight gain at an early stage is found to be the most critical factor for offspring metabolic outcomes along with the previous negative outcomes. Moreover, it has been demonstrated that even a late screening (>28 weeks) could provide substantial benefits for the patients by providing the appropriate management [11].

Due to the lack of criteria for early screening, many health practitioners prefer to adopt multiple screening variations when assessing GDM [12]. There have been studies trying to apply the standard guidelines for early screening but achieving only controversial and contradictory results [10,13]. Other studies have tried to implement other screening tests rather than OGTT such as HbA1c, random plasma glucose (RPG) or fasting plasma glucose (FPG) and have acquired promising results in the early detection of GDM with the potential of renewing the current guidelines [14-21]. Finally, previous studies demonstrated that performing early (<24 weeks) or late (>28 weeks) screening tests are beneficial for patients [22-24], especially for those women with a high-risk profile such as having obesity, and older age. This study aimed to determine whether performing early or late screening test for GDM impacts in outcomes, either positively or negatively in patients diagnosed with the disease.

## Methods

**Study design and setting:** the methods for this cross-sectional study have been published elsewhere [25], but briefly we invited patients who attended the outpatient clinic from December 1^st^, 2017 to February 31^st^, 2020 of family medicine or pregnancy clinic at the General Regional Hospital No. 6 “Lic. Ignacio Garcia Tellez” of the Social Security Mexican Institute in Madero City, Tamaulipas, Mexico. This protocol was not submitted to an ethics committee, as the OGTT is part of the usual care of pregnant women; however, patient´s consent to participate and use the data for this protocol was agreed upon verbally. Sample size estimation was not performed, as the sample was selected based on convenience.

**Study population:** we included all pregnant patients who agreed to participate regardless of their pregnancy trimester. We asked the patients that were interested to participate in our study to complete a survey form which included general information as name, age, and a list of risk factors for GDM (e.g. overweight/obesity, family history of T2DM, multiparity, hypertension, dyslipidemia, presence of acanthosis nigricans, polycystic ovary syndrome, and weight gain >20kg in previous pregnancies. All participants that had >3 factor for GDM were invited to participate in the study. We excluded all patients with a known diagnosis of any type of pre-gestational diabetes, patients who already had an established diagnosis of GDM, patients who did not complete the OGTT testing, those who did not complete the baseline survey, and patients that did not have a complete health electronic record. After the participant´s acceptance to be included in the study, clinical anamnesis was made for every patient, including name, age, family history of DM, comorbidities, gestational age, weight change during the pregnancy, previous pregnancies, and previous obstetric complications. Anthropometric measurements were taken, including weight, height, and BMI. Treatment and dose adjustments for each patient were decided by the primary care physician following current guidelines [5,26], follow-up consults were decided depending on the participant glycemic control, participants in good control (defined as fasting glucose < 95mg/dl, 1-hour postprandial glucose < 140mg/dl, and 2-hrs postprandial glycemia < 120mg/dl were evaluated every 4-weeks, and patients outside these glycemic goals had a medical appointment scheduled every 2-weeks until glycemic control was achieved, and until the end of the pregnancy.

**Testing:** all patients underwent OGTT testing with a load of 75 grams. The OGTT was made at enrollment, and they were made in a case consecutive manner regardless of their gestational age. The diagnosis was labeled according to the criteria proposed by the IADPSG in 2010 and according to the HAPO study (a positive value in any of the three measurements: >92mg/dL at fasting, >180mg/dL at one hour, or >153mg/dL at 2 hours) [27]. Patients with normal results in all three measurements were classified as controls and were kept under surveillance with their primary care physician. Patients with abnormal results in any of the three measurements were diagnosed with GDM. We defined as “standard time” the group that was assessed between week 24-28 of pregnancy as stated by the current guidelines [28], “early screening” was defined as the group that made the OGTT before week 24 of pregnancy, and “late screening” was defined as the group that made the OGTT after week 28 of pregnancy.

**Variables:** the primary aim of this study was to estimate the proportion of patients diagnosed with GDM using the IADPSG criteria regardless of the gestational age. Secondary aims were: 1) to estimate the proportion of patients diagnosed with GDM during the first and third trimester of pregnancy and the proportion of those diagnosed during the second trimester; 2) to identify risk factors for developing GDM in any of the three trimesters and then stratify by trimester in which they were diagnosed; 3) to assess for difference in maternal (defined as frequency of preeclampsia, urinary trach infections/cervicovaginitis, premature rupture of membranes, induction, eutocic birth, dystocic birth, caesarean section, and polyhydramnios) and fetal outcomes (defined as birth weight inkg, classified as normal weight/underweight, and macrosomic product, height in cm, frequency of hypoglycemia events, respiratory distress syndrome, fetal distress, need of supplementary oxygen, intubation/ventilatory support, and jaudince) among patients with and without GDM in any of the three trimesters and then stratified by the trimester in which they were diagnosed.

**Statistical analyses:** numerical variables are summarized as means and standard deviations. Categorical variables are summarized as frequencies and percentages. We used Pearson´s Chi-square test to identify associations between the diagnoses of GDM and categorical baseline characteristics, and between diagnosis of GDM and categorical maternal and neonatal complications. To identify the associations between diagnosis of GDM and numerical baseline characteristics, and between diagnosis of GDM and numerical maternal (frequency of preeclampsia, urinary tract infections/cervicovaginitis, premature rupture of membranes, induction, eutocic birth, dystocic birth, caesarean section, and polyhydramnios) and neonatal complications (birth weight, classified as normal weight/underweight, and macrosomic product, height in cm, frequency of hypoglycemia events, respiratory distress syndrome, fetal distress, need of supplementary oxygen, intubation/ventilatory support, and jaundice) we used Student´s test for independent samples. We fitted a series of univariable models to measure the association between individual risk factors (age, overweight/obesity), family history of DM, obstetric complications, insulin resistance, weight gain >20kg, hypertension, hyperlipidemia, and multiparity) and GDM. Then, we fitted a multivariable logistic regression with those variables with significant associations in the univariable models, using a statistical significance threshold of a p value <0.05. We used the statistical package SPSS version 25 for Mac OS (IBM, Armonk, NY, USA) for all statistical analyses.

## Results

**General characteristics:** a total of 803 patients were included. The mean age of the population was 29.89 Â± 5.73, and a mean gestational age at the end of pregnancy of 37.63 + 4.4 weeks. A total of 292/803 (36.4%) of the patients were diagnosed with GDM using the IADPSG criteria. Of these, 219/292 (75%) had impaired fasting blood glucose, 158/292 (54.1%) had impaired oral glucose tolerance at 60 minutes, and 144/292 (49.3%) at 120 minutes. General characteristics are shown in Table 1.

**Table 1:** baseline clinical and biochemical characteristics of the included pregnant participants

	Total (n= 803)			Early Test (<24 gestational age) (n= 286)			Standard test (24-28 gestational age) (n= 232)			Late test (>28 gestational age) (n= 285)		
	Controls (n= 511)	GDM (n= 292)	p value	Controls (n= 184)	GDM (n= 102)	p value	Controls (n= 153)	GDM (n= 79)	p value	Controls (n= 174)	GDM (n= 111)	p value
**General characteristics**												
Age, mean ± SD	29.04 ± 5.78	31.39 ± 5.33	<.001*	29.43 ± 5.64	32.36 ± 4.91	<.001*	29.50 ± 5.68	31.29 ± 5.60	.023*	28.23 ± 5.96	30.65 ± 5.44	.001*
BMI, mean ± SD	29.67 ± 6.05	32.59 ± 5.86	<.001*	29.33 ± 5.51	32.55 ± 6.05	<.001*	29.47 ± 6.05	32.45 ± 6.22	.001*	30.20 ± 6.57	32.72 ± 5.47	.001*
Normal BMI normal, n (%)	110 (21.5)	25 (8.6)	<.001*	33 (17.9)	8 (7.8)	<.001*	37 (24.2)	7 (8.9)	.008*	40 (23)	10 (9)	<.001*
Overweight BMI, n (%)	183 (35.8)	72 (24.7)		84 (45.7)	27 (26.5)		48 (31.4)	23 (29.1)		51 (29.3)	22 (19.8)	
Obesity BMI, n (%)	218 (42.7)	195 (66.8)		67 (36.4)	67 (65.7)		68 (44.4)	49 (62)		83 (47.7)	79 (71.2)	
**Oral glucose tolerance test values**												
OGTT Fasting positive test for GDM, n (%) (n=799)	0 (0)	219 (75)	-	0 (0)	82 (80.4)	-	0 (0)	65 (82.3)	-	0 (0)	72 (64.9)	-
OGTT Fasting glucose values (mg/dl), mean ± SD (n=799)	80.62 ± 6.27	97.95 ± 13.97	<.001*	81.88 ± 6.04	98.90 ± 13.88	<.001*	80.90 ± 6.35	99.21 ± 13.69	<.001*	79.75 ± 6.27	96.19 ± 14.19	<.001*

**Diagnosis of GDM stratified by screening time:** after stratifying the population by screening time, a total of 232/803 (28.9%) underwent screening in the standard time, 286/803 (35.6%) underwent early screening, and 285/803 (35.5%) underwent late screening. From those that underwent standard screening, a total of 79/232 (34%) were diagnosed with GDM. Similarly, 102/286 (35.6%) and 111/285 (38.9%) were diagnosed with GDM, in the early and late screening times, respectively. Characteristics stratified by screening time are shown in Table 1.1.

**Table 1.1:** baseline clinical and biochemical characteristics of the included pregnant participants

	Total (n= 803)			Early Test (<24 gestational age) (n= 286)			Standard test (24-28 gestational age) (n= 232)			Late test (>28 gestational age) (n= 285)		
	Controls (n= 511)	GDM (n= 292)	p value	Controls (n= 184)	GDM (n= 102)	p value	Controls (n= 153)	GDM (n= 79)	p value	Controls (n= 174)	GDM (n= 111)	p value
**Oral glucose tolerance test values**												
OGTT 60 min positive test, n (%) (n=799)	0 (0)	158 (54.1)	-<.001*-	0 (0)	48 (47.1)	-<.001*-	0 (0)	36 (45.6)	-<.001*-	0 (0)	67 (6.4)	-<.001*-
OGTT 60 min glucose values (mg(dl), mean ± SD (n=798)	131.29 ± 23.82	184.31 ± 37.14		126.74 ± 24.69	179.11 ± 39.86		126.93 ± 22.46	185.09 ± 37.03		140.04 ± 21.64	188.54 ± 34.26	
OGTT 120 min positive test, n (%) (n=796)	0 (0)	144 (49.3)		0 (0)	40 (39.2)		0 (0)	41 (51.9)		0 (0)	63 (56.8)	
OGTT 120 min glucose values (mg/dl), mean ± SD (n=796)	111.32 ± 19.14	152.09 ± 37.55	<.001*	109.43 ± 20.00	145.46 ± 34.46	<.001*	111.44 ± 19.31	152.03 ± 37.46	<.001*	113.24 ± 17.93	158.16 ± 39.55	<.001*

* = p=<0.05 (statistically significant), GDM= gestational diabetes mellitus, SD= standard deviation, BMI= body mass index, OGTT: oral glucose tolerance test.

**Risk factors for developing GDM by screening time:** all of the risk factors frequencies analyzed in the total population are described in Annex 1. The risk factors for GDM in the total population that had an association in the univariable and multivariable logistic regression analysis were age > 30y, overweight/obesity, and family history of diabetes (Annex 2). In Table 2, we described the analysis made by screening time, in the standard screening group, age > 30 years, being overweight/obesity, and hypertension were associated with GDM in the univariable analysis. However, only age >30 years aOR [1.78 (95% CI 1.10-3.14); p=.046] and overweight/obesity aOR [2.93 (95% CI 1.22-6.99); p=.015] remained significant in the multivariable logistic regression analysis. While in the early screening group, the univariable analysis showed risk factors with statistical difference, including maternal age >30 years, overweight/obesity, family history of DM, history of obstetrics complications, hypertension, and hyperlipidemia. However, in the multivariable logistic regression analysis, only maternal age >30 years aOR [3.04 (95% CI 1.79-5.15); p=.001] and family history of DM aOR [2.57 (95% CI 1.36-4.84); p=.003] remained significant. In the late screening group, age >30 years, overweight/obesity, family history of DM, and multiparity were associated with GDM in the univariable analysis, but only age >30 years aOR [1.81 (95% CI 1.11-2.94); p=.017] and family history of DM aOR [1.92 (95% CI 1.08-3.40); p=.025] maintained the association in the multivariable logistic regression analysis.

**Table 2:** univariable and multivariable logistic regression analysis of predictors of gestational diabetes mellitus by screening time

	Univariable		Multivariable	
	OR (CI 95%)	p value	Adjusted Odds Ratio (CI 95%)	p value
**Early screening time (<24 weeks)**				
Age> 30	3.06 (1.82-5.14)	<.001*	3.04 (1.79-5.15)	<.001
Overweight/Obesity	2.56 (1.13-5.79)	.023*	-	-
Family health history of DM	2.60 (1.40-4.81)	.002*	2.57 (1.36-4.84)	.003
Obstetric problems	1.91 (1.16-3.13)	.010*	-	-
Insulin resistance	1.34 (.82-2.18)	.231	-	-
Weight Gain (>20 kg)	1.00 (.50-1.98)	.994	-	-
Hypertension	2.46 (1.13-5.35)	.023*	-	-
Hyperlipidemia	1.71 (1.03-2.85)	.036*	-	-
Multiparous	1.36 (.84-2.18)	.202	-	-
**Standard screening time (24-28 weeks)**				
Age>30	1.91 (1.14-3.47)	.015*	1.78 (1.10-3.14)	.046
Overweight/Obesity	3.28 (1.38-7.75)	.007*	2.93 (1.22-6.99)	.015
Family health history of DM	1.36 (.76-2.45)	.297	-	-
Obstetric problems	1.65 (.93-2.93)	.086	-	-
Insulin resistance	1.68 (.97-2.90)	.064	-	-
Weight Gain (>20 kg)	1.02 (.43-2.42)	.949	-	-
Hypertension	3.02 (1.10-8.27)	.031*	-	-
Hyperlipidemia	.67 (.29-1.54)	.348	-	-
Multiparous	1.02 (.58-1.79)	.935	-	-
**Late screening time (>28 weeks)**				
Age > 30	1.77 (1.09-2.86)	.020*	1.81 (1.11-2.94)	.017*
Family health history of DM	1.87 (1.06-3.92)	.030*	1.92 (1.08-3.40)	.025*
Overweight/Obesity	3.01 (1.43-6.31)	.003*	-	-
**Late screening time**				
Obstetric problems	1.11 (.65-1.89)	.701	-	-
Insulin resistance	1.40 (.87-2.25)	.157	-	-
Weight gain>20 kg	1.40 (.64-3.08)	.394	-	-
Hypertension	1.00 (.42-2.41)	.985	-	-
Hyperlipidemia	1.03 (.41-2.57)	.950	-	-
Multiparous	1.88 (1.14-3.09)	.012*	-	-

* Statistically significant. OR= odds ratio; CI= confidence interval

***Maternal outcomes (population description and comparison between standard vs. early vs. late screening):*** patients on early screening had a higher proportion of patients with GDM who developed preeclampsia (p=.025) compared with the group without GDM. There were no significant differences in the other outcomes. However, there were significant differences in the distribution of the proportion of patients with respect to the delivery method between controls and cases. For instance, more GDM participants required cesarean section 147/258 (83.1%) vs. 187/177 (72.5%) control; p=.037] and less experienced an eutocic labor 26/177 (14.7%) vs 62 (24%), respectively; p=.037]. The remaining maternal outcomes are shown in Table 3. In the univariable analysis between risk factors associated with GDM and caesarean section, overweight/obesity, and multiparous were significant, and maintained it in the multivariable logistic regression analysis with an aOR [1.90 (95% CI 1.08-3.35);p=.026], and an aOR [0.57 (95% CI 0.36-0.91);p=.017] respectively (Annex 3). Univariable and multivariable logistic regression analysis regarding preeclampsia and gestational age and their respective association with GDM risk factors are detailed in supplementary Annex 4, Annex 5.

**Table 3:** maternal outcomes by screening time

	Total (n= 803)			Early Test (<24 gestational age) (n= 286)			Standard test (24-28 gestational age) (n= 232)			Late test (>28 gestational age) (n= 285)		
	Controls (n= 511)	GDM (n= 292)	p value	Controls (n= 184)	GDM (n= 102)	p value	Controls (n= 153)	GDM (n= 79)	p value	Controls (n= 174)	GDM (n= 111)	p value
Preeclampsia, n (%) (n= 438)	15 (5.8)	17 (9.4)	.159	3 (4.2)	10 (15.6)	.025*	3 (3.7)	4 (8.5)	.242	9 (8.7)	3 (4.3)	.265
Gestational age, mean ± SD (n=441)	38.03 ± 2.97	37.06 ± 5.89	.043*	37.51 ± 4.97	35.18 ± 9.58	.076	37.91 ± 1.95	38.31 ± 1.09	.200	38.46 ± 1.46	37.92 ± 1.15	.011*
UTI/Cervicovaginitis, n (%) (n= 438)	52 (20.2)	43 (23.8)	.451	14 (19.7)	14 (21.9)	.758	19 (23.2)	7 (14.9)	.259	19 (18.3)	22 (31.4)	.072
PROM, n (%) (n= 438)	13 (5.1)	13 (7.2)	.354	3 (4.2)	5 (7.8)	.378	6 (7.3)	0 (0)	.058	4 (3.8)	8 (11.4)	.053
Induction, n (%) (n= 438)	29 (11.3)	20 (11)	.939	6 (8.5)	5 (7.8)	.892	6 (7.3)	4 (8.5)	.807	17 (16.3)	11 (15.7)	.911
Eutocic, n (%) (n= 435)	62 (24)	26 (14.7)	.037*	10 (14.5)	12 (20.3)	.585	21 (25)	4 (8.3)	.050	31 (29.5)	10 (14.3)	.061
Dystocic, n (%) (n= 435)	9 (3.5)	4 (2.3)		4 (5.8)	2 (3.4)		3 (3.6)	1 (2.1)		2 (1.9)	1 (1.4)	
Caesarean section, n (%) (n= 435)	187 (72.5)	147 (83.1)		55 (79.7)	45 (76.3)		60 (71.4)	43 (89.6)		72 (68.6)	59 (84.3)	
Polyhydramnios, n (%) (n= 436)	9 (3.5)	2 (1.1)	.118	5 (7.1)	1 (1.6)	.123	4 (4.8)	1 (2.1)	.431	0 (0)	0 (0)	-
Cholesterol, mean ± SD (n= 768)	216.80 ± 48.26	210.11 ± 46.66	.062	189.97 ± 41.85	187.81 ± 35.12	.663	229.06 ± 41.25	220.57 ± 46.63	.166	235.77 ± 47.47	223.26 ± 48.75	.038*
Triglycerides, mean ± SD (n=768)	191.23 ± 65.47	217.80 ± 79.39	<.001*	152.26 ± 51.26	182.84 ± 69.16	<.001*	200.63 ± 60.50	206.45 ± 69.39	.518	225.95 ± 61.19	258.36 ± 77.50	<.001*

UTI: urinary tract infection, PROM: premature rupture of membranes

***Neonatal outcomes (population description and comparison between standard vs. early vs. late screening):*** we found that newborns of women with GDM of the late screening group were larger compared to the women without GDM (p=0.016), no other significant differences were found in the neonatal outcomes between groups (Table 4). In the univariable analyses between neonatal height and risk factors for GDM, hyperlipidemia and weight gain were significant, and maintained it in the multivariable logistic regression analysis with an aOR [1.37 (95% CI 0.56-2.19);p=.001], and aOR [0.95 (95% CI 0.04-1.86);p=.041] respectively (Annex 6).

**Table 4:** neonatal outcomes by screening time

	Total (n= 803)			Early Test (<24 gestational age) (n= 286)			Standard test (24-28 gestational age) (n= 232)			Late test (>28 gestational age) (n= 285)		
	Controls (n= 511)	GDM (n= 292)	p value	Controls (n= 184)	GDM (n= 102)	p value	Controls (n= 153)	GDM (n= 79)	p value	Controls (n= 174)	GDM (n= 111)	p value
Weight, mean ± SD (n= 437)	3.20 ± .52	3.22 ± .55	.715	3.24 ± .53	3.08 ± .54	.092	3.08 ± .49	3.19 ± .49	.240	3.28 ± .53	3.38 ± .55	.234
Normal weight, n (%) (n= 437)	224 (86.8)	153 (85.5)	.920	61 (87.1)	56 (88.9)	.232	73 (88)	38 (80.9)	.431	90 (85.7)	59 (85.5)	.575
Underweigh, n (%) (n= 437)	18 (7)	14 (7.8)		4 (5.7)	6 (9.5)		8 (9.6)	6 (12.8)		6 (5.7)	2 (2.9)	
Macrosomic, n (%) (n= 437)	16 (6.2)	12 (6.7)		5 (7.1)	1 (1.6)		2 (2.4)	3 (6.4)		9 (8.6)	8 (11.6)	
Height, mean ± SD (n= 433)	49.67 ± 2.71	49.64 ± 3.71	.912	49.63 ± 3.04	48.48 ± 4.22	.073	49.62 ± 2.55	49.36 ± 2.28	.565	49.74 ± 2.63	50.91 ± 3.64	.016*
Hypoglycemia, n (%) (n= 436)	3 (1.1)	1 (.5)	.884	2 (2.9)	1 (1.6)	.622	1 (1.2)	0 (0)	.445	0 (0)	0 (0)	-
RDS, n (%) (n= 437)	9 (3.5)	11 (6.1)	.199	4 (5.7)	6 (9.5)	.405	3 (3.6)	2 (4.2)	.874	2 (1.9)	3 (4.3)	.351
Acute fetal distress, n (%) (n= 437)	11 (4.3)	5 (2.8)	.410	3 (4.3)	2 (3.2)	.737	4 (4.8)	1 (2.1)	.431	4 (3.8)	2 (2.9)	.739
Oxygen, n (%) (n= 436)	17 (6.6)	17 (9.5)	.270	4 (5.7)	7 (11.1)	.259	6 (7.2)	2 (4.2)	.481	7 (6.7)	8 (11.8)	.253
Intubation, n (%) (n= 436)	3 (1.2)	4 (2.2)	.383	0 (0)	2 (3.2)	.133	1 (1.2)	1 (2.1)	.693	2 (1.9)	1 (1.5)	.825
Jaundice, n (%) (n= 436)	1 (0.4)	2 (1.1)	.366	1 (1.4)	0 (0)	.341	0 (0)	2 (4.2)	.061	0 (0)	0 (0)	-

* = p value < 0.05, GDM= gestational diabetes mellitus, RDS: respiratory distress syndrome

## Discussion

In our study population, there was not a significant difference in the frequency of GDM diagnosis, regardless of the pregnancy trimester in which the test was performed, when using the IADPSG criteria for diagnosis. Significant risk factors for GDM diagnosis regardless gestational age were identified through a univariable and multivariable regression model: maternal age >30 years. Beside this, in the standard screening group the presence of maternal overweight and obesity was defined as a risk factor; lastly, in both early and late screening group family history of diabetes mellitus was identified as a risk factor.

In a prospective cohort study published in 2020, it was also found that there was not a significant association between early GDM screening and maternal or neonatal outcomes. Unlike our study, most subjects in this cohort turned out to be healthy (84%) and advanced maternal age was not found as a significant risk factor for early GDM diagnosis. As a methodological difference, all subjects were submitted to early, standard, and late GDM screening [29]. However, another retrospective cohort found that early GDM screening had a significant influence over maternal and neonatal outcomes, mainly: cesarean section, macrosomia, large for gestational age, neonatal intensive care unit admission, neonatal respiratory distress syndrome, and Apgar score at 1-minute <7. Nevertheless, they also stated that advanced maternal age was a relevant risk factor for early and late GDM [30]. In another study the early screening of GDM helped to reduce the GWG during pregnancy. Despite this benefit, there was not any significant improvement or change in the maternal and neonatal outcomes [31]. Moreover, another retrospective cohort study carried out 2020, showed in its results that patients that developed GDM at an early stage were significantly propense to need caesarean section and neonates were admitted with greater frequency to the neonatal intensive care unit [32]. Lastly, Simmons *et al*. in a RCT [33] stated that in a group of patients with GDM (diagnosed before 20-weeks) treated with dietary advice, and initiation/intensification of pharmacotherapy had a modestly, yet significant lower incidence of a composite of adverse neonatal outcomes compared to a no early treatment group (diagnose made after 20-weeks); however, it is important to specify that the trial population was composed of patients with higher-risk for hyperglycemia.

The differences in the outcomes between studies could be explain by several factors, mainly for the heterogeneity of the definition of “early screening”, and “late screening” with all studies using different gestational age for their definition, the differences in definition of “standard care” in which each medical unit have their own treating and follow-up policies attending patients with GDM, as the initiation-adjustment of pharmacological treatment besides the lifestyle changes and the follow-up consults were not specified or were different between studies, and lastly; the differences between the risk factors of the participants of each study at baseline, considering ethnicity, and a higher BMI at inclusion in our study could explain the differences in the outcomes reported between studies [29-33].

Although several studies have not been able to show benefits of early or late screening times in terms of fetal or maternal outcomes in a consistent manner [29-33], our study adds new information regarding this topic with potential clinical implications. Early and late screening should not be used (as of this moment) as a general screening strategy as we did not see a difference regarding the frequency of diagnosis of GDM; however, we hypothesize it may be useful in specific situations like in a high-risk patient defined as a woman with family history of diabetes, maternal age >30 years and with overweight/obesity, it may be also useful with certain populations like patients with a difficult access to continuous medical appointments as may happen in rural population or in developing countries, patients with a poor adherence to their medical appointments where performing the OGTT regardless of the gestational time may help to make a diagnosis and give proper treatment. However, larger and prospective studies are needed to evaluate the usefulness of these strategies in high-risk profile populations.

Our study has several limitations; first, the lack of randomization in the present study, even after adjusting for several confounders in our multivariable regression analyses, may introduce some bias due to confusion in our results, and we did not include the specific treatment per group of patients. As strengths, the consecutive cases sampling method that was used in this study also reduces selection bias and ensures that the analyzed sample is representative of the population from which it was withdrawn.

## Conclusion

Our results suggest a similar proportion of patients are diagnosed with GDM when comparing the early, standard and late screening times using the IADPSG criteria. Risk factors like maternal age >30 years, family history of diabetes and overweight/obesity should be actively interrogated as they may be associated with GDM diagnosis. Lastly, early or late screening times did not provide any additional benefits on maternal and neonatal outcomes in our population.

### What is known about this topic

- Gestational diabetes is associated with multiple negative maternal and neonatal outcomes;
- There is a growing interest in assessing the impact of early and late gestational diabetes screening on maternal and neonatal outcomes;
- New strategies are needed to improve maternal and neonatal outcomes in patients with GDM.

### What this study adds

- We did not find a difference in neonatal outcomes between the early and late screening groups;
- Maternal age >30 years was associated as a risk factor for GDM, regardless of the gestational age when the diagnosis was made;
- Family history of diabetes was associated as a risk factor for GDM in patients who underwent the early and the late screening.

## Annexes

**Annex 1**: risk factors for gestational diabetes mellitus (PDF-76KB)

**Annex 2**: univariable and multivariable logistic regression analysis of predictive factors of gestational diabetes mellitus in the total population (PDF-78KB)

**Annex 3**: univariable and multivariable logistic regression analysis regarding caesarean section as an associated risk factor for gestational diabetes mellitus in maternal outcomes (PDF-74KB)

**Annex 4**: univariable and multivariable logistic regression analysis regarding preeclampsia as an associated risk factor for gestational diabetes mellitus in maternal outcomes (PDF-75KB)

**Annex 5**: univariable and multivariable logistic regression analysis regarding gestational age as an associated risk factor for gestational diabetes mellitus in maternal outcomes (PDF-78KB)

**Annex 6**: univariable and multivariable logistic regression analysis regarding neonatal height as an associated risk factor for gestational diabetes mellitus in neonatal outcomes (PDF-73KB)
